# Parents of children with autism spectrum disorder quality of life in Jordan: a comparative study

**DOI:** 10.2144/fsoa-2023-0096

**Published:** 2024-05-15

**Authors:** Abedallah Kasem, Sawsan Abuhammad, Naemah Jamal

**Affiliations:** 1 Department of Maternal and Child Health, Faculty of Nursing, Jordan University of Science and Technology, P.O. Box 3030, Irbid 22110, Jordan

**Keywords:** ASD, autism, child, chronic disease, family, healthcare, life, nurse, parenting, QoL

## Abstract

**Aim:** This study aimed to assess the quality of life (QoL) of parents of children with autism spectrum disorder (ASD) in Jordan and its associated factors. **Methods:** This comparative study was conducted among parents of children with ASD and non-ASD. Data collection took four setting areas that include three centers for autism in three different municipalities and the control group was collected using social media. A convenience sample of 242 parents agreed to complete a QoL questionnaire. **Results:** Parents of children with ASD in Jordan have poor QoL across the five domains of QoL in compare with parents with non-ASD child. Factors such as gender, level of education, living condition and employment status were found impacting QoL.

## Abstract (plain language summary)

Factors such as gender, level of education, living condition and employment status were found impacting quality of life (QoL) of autism spectrum disorder (ASD) parents.

Researchers have indicated that parents of children with ASD suffer from poor QoL.

Counseling would assist the parents to overcome the negative thoughts associated with the challenges they face in caring for children with ASD.

Autism spectrum disorder (ASD) is a complex neurodevelopmental disorder that involves persistent deficits in reciprocal social interaction and communication, restricted/repetitive behaviors and stereotyped interests. Currently, there is a lack of an available cure [^1-3^]. In recent times, the prevalence rate of autism among the global population has dramatically increased [4]. The estimated increase among the population is about 6 per 1000 children yearly [5].

Autism became a subject of interest in the Arab world during the late 1990s and the field of child psychiatry is relatively new [6]. In Jordan, among a clinical sample of children with global developmental delay, 5.2% were found to have autism [7]. However, it is not known if autosomal recessive disorders contribute to the incidence of autism in Jordan [7]. Nonetheless, it is known that an increase in the number of children with autism will increase the burden of care on both the healthcare service and the family. For instance, a study on 181 Brazilian families noted that children with ASD need a high level of care and assistance in their daily living activities [8]. Moreover, the study reported that parents/parents often find it difficult to integrate regular family activities and household chores with the care they need to provide for their children with ASD. Specifically, the parents encountered a moderate to severe burden of care and experienced higher levels of parenting stress, reduction in their activities, health and psychological problems and lower quality of life (QoL).

QoL is a multidimensional concept that includes physical, psychological and social domains of health [^9-11^]. Studies conducted in different parts of the world such as America [12], Europe [13], India [14], Taiwan [15] and Australia [16] have reported that physical, psychological, social and environmental QoL among parents and parents of children with ASD is low, and that they are susceptible to psychological disorders including depression, anxiety and stress. In Qatar, parents in an autism group ranked lower in general health domains as compared with the control group [17]. It has also been shown that daily encounters with children with ASD lead to mental discomfort and a range of associated negative lifestyle outcomes among parents [18]. Authors [^19-22^] revealed that some of the triggers of distress among parents include inability to meet the child's social wellbeing needs, financial difficulties, inability of parents to express themselves, difficulties when participating in social gathering activities, as well as worries and anxiety.

Researchers have indicated that parents of children with ASD suffer from poor QoL due to the need to give additional care and attention to their children [18], and in giving this care parents fail to attend to their daily economic activities [23]. Consequently, the psychological health and the overall wellbeing of the parents gradually deteriorate [23,24]. Further, it has been hypothesized that families who provide support and care to children with various forms of disabilities and chronic diseases often face one or more forms of hardship in their life, which might affect the quality of their lives [25]. However, this issue has not been given much consideration in the Jordanian context. In fact, there is a critical research gap in respect of local studies in Jordan and the surrounding nations [26,27].

In Jordan, little attention has been paid to the needs of parents of children with ASD and these parents receive little support on how to deal with such a challenge [26]. In fact, the lack of studies means that there is scant knowledge on Jordanian parents' perspectives of the level of their QoL while caring for a child diagnosed with autism. Yet, from the limited number of studies published to date, it seems that Jordanian parents have reported stressful experiences including stigma, inadequate knowledge of ASD, lack of professional support services, delay in diagnosis for their children and concerns about the educational endeavors of their children [^28-30^]. Therefore, the present study aimed to assess QoL among parents of children with ASD in Jordan and its associated factors to add to knowledge.

## Methods

### Study design & participants

This comparative study was conducted among parents of children with ASD and non-ASD. Data collection took four setting areas that include three centers for autism in three different municipalities and the control group was collected using social media. The Autism Association of Jordan is in Amman, the capital city of Jordan. This association was established in 2014 as a nonprofit organization. It provides care and services to a total of 70 parents of children diagnosed with autism.

▪ The Sanabel Center for Autism which is in Al-salat city, in Balqa Municipality. This center was founded in 2017 as a charitable social institution to provide services to 40 Jordanian children with ASD;
▪ The Wasan Center in Irbid Municipality in the north of Jordan. This center is a privately run organization that provides services and care to 35 parents of children diagnosed with autism;
▪ The control group was collected from the social media such as Facebook, WhatsApp and Instagram.

### Data collection procedure

Parents of children with ASDs were recruited from the three above mentioned targeted settings. To take part in the study, the participants had to meet the following inclusion criteria: living in an urban or rural area registered at one of the chosen settings; providing direct care to a child aged 3–16 years and diagnosed with ASD; and speaking Arabic. For the control group, parents with a child aged 3–16 years old and speaking Arabic were qualified for the study.

### Ethical consideration

Ethical approval for the study was obtained from the Jordan University of Science and Technology (#23-2020) and the ethical committees of the three settings. A convenience sample of parents who had a child with ASD was approached by contacting the managers of the three centers, who facilitated reaching out to parents of children with autism through their WhatsApp numbers and e-mail addresses. The other 121, for the control group was approached by the researchers. Consequently, 242 parents were invited via WhatsApp and e-mail to complete QoL questionnaire adapted from Mansi and Kazem [31]. The confidentiality of the respondents was ensured. They were informed of their right to participate voluntarily and to withdraw from the study at any time during the data collection period without any penalties, risks, or physical or psychological threats or harm. They were also informed that their collected responses would be used for study purposes only. Before completing the questionnaire, electronic informed consent was obtained from each respondent who had agreed to participate, and the researcher was available to answer any questions they had about the questionnaire. All the completed questionnaires were kept in a secure file cabinet with access only available to the researchers.

### Instrument

The QoL scale, developed by Mansi and Kazem [31], was used to evaluate the QoL among parents of children with ASD. The adapted questionnaire consisted of two main parts. The first part was concerned with the demographic characteristics and asked questions about age, gender, educational level, marital status, living condition and employment status. The second part of the questionnaire was mainly focused on the QoL scale developed by Mansi and Kazem [31]. The QoL scale consisted of 50 items grouped into subscales focused on the following five dimensions: general health of the parents, family and society and their support to the parents, emotions that parents were carried out, mental health of the parents and time management of the family to carry out all related tasks. Parents' general health is essential to their capacity to carry out their obligations effectively. Parents who are in good physical shape can be more engaged and active in their children's lives. Maintaining good health requires regular physical activity, a well-balanced diet and enough sleep. Accessibility to healthcare services, wellness programs and chronic illness management are all important considerations. Family and societal support includes the support network that parents have is essential. Parenthood can be made easier with the assistance of extended friends, relatives and local networks. Childcare aid, counsel and psychological assistance are all examples of family support. The support of society can be seen in the form of accessible and cheap childcare facilities, parental leave legislation and community organizations that provide services and assistance. Emotions that parents carry which related to parents frequently experience a variety of feelings. Parenthood can bring joy, love and happiness, but it can also bring anxiety, fear, frustration and shame. Emotions might vary based on the age of the child, phases of development and household conditions. Parents can cope with these feelings by discussing openly and getting emotional support. Parental mental health is related to psychological well-being of parents which is really crucial. Parenting can be difficult, and it is natural for one to become overwhelmed at times. Postpartum sadness, anxiety and burnout can all have an impact on parents' mental health. It is critical for parents to seek assistance when it is required, whether through counseling, psychotherapy or support groups. It is critical that we minimize the stigma associated with mental health in order for families to feel comfortable getting care. Time management in families is critical for parents to organize their time wisely in order to fulfill their duties. Setting objectives, developing calendars and allocating chores within the family are all part of this. Effective time management allows parents to have meaningful time with their children, practice self-care and meet other duties such as employment or housework. The five dimensions were measured by asking ten questions about each, the responses to which were measured on a five-point Likert scale (totally agree, agree, neutral, disagree, totally disagree). The overall QoL was dichotomized using the median as the cutoff point. The internal consistency/reliability of the QoL scale was (0.74). The back translation method was applied to ensure that the original (English) and the second language (Arabic) version of the instrument measured the same concepts [32]. The face validity and content validity of the translated version of the instrument was checked and evaluated by three panels of experts.

### Data analysis

The SPSS program was used to analyze the data. A five-point scale was used to obtain the responses. A linear regression analysis was conducted to determine the factors associated with low QoL. A p-value of 0.05 was considered statistically significant. Means, medians, standard deviations, frequencies and percentages were reported as appropriate to the descriptive purposes. The Statistical Package for the Social Sciences, version 23.0 was used for statistical analysis.

## Results

### Sociodemographic characteristics of parents of children with autism

This study recruited 242 parents. This first group consist of 121 parents of children diagnosed with autism and 121 none diagnosed with autism parents were included in the study. Their characteristics were presented in Table 1. There were no significant differences in the characteristics of the participants.

**Table 1.** Sociodemographic characteristics of parents of children with autism newborns (n = 241).

Variable	Category	ASD	Non-ASD
Age (years)	–	34.69/5.499	31.00 (7.035)
Gender	Male Female	41 (33.9%) 80 (66.1%)	29 (24%) 92 (76%)
Education level	Primary Secondary University degree Tertiary	2 (1.7%) 64 (52.9%) 51 (42.1%) 4 (3.3%)	5 (4.1%) 61 (50.4%) 52 (43.0%) 3 (2.5%)
Marital status	Single Married Divorced	1 (0.8%) 116 (95.9%) 4 (3.3%)	10 (8.3%) 102 (84.1) 19 (7.4%)
Living condition	Living alone Living with people	3 (2.5%) 118 (97.5%)	3 (2.5%) 118 (97.5%)
Employment status	Formal employment Informal employment Unemployed	16 (13.2 %) 56 (46.3 %) 49 (40.5%)	62 (51.2%) 32 (26.4%) 27 (22.3%)

ASD: Autism spectrum disorder.

### Comparison in five dimensions of quality of life for autism spectrum disorder & parents who have do not have a child with autism spectrum disorder

Parents of children diagnosed with ASD scored in their QoL lower in all dimensions of life compared with non-parents who have ASD child (p = 0.001) as shown in Table 2. The highest difference was quality of mental health for ASD (M = 21.73, SD = 2.217) compared with non-ASD (M = 27.9421, SD = 4.28427; p = 00.001). The lowest score was in public health quality for ASD (M = 17.74, SD = 3.061) and non-ASD (M = 23.9587, SD = 4.27082; p = 0.012).

**Table 2.** Distribution of the scores of parents of children with autism for the five domains of quality of life.

Item	Mean	Std. Deviation	Mean	Std. Deviation	p-value
Quality of mental health	21.73	2.217	27.9421	4.28427	0.001
Quality of emotions	21.06	2.367	27.56	3.96239	0.011
Quality of time management	20.35	2.452	27.8595	5.06344	0.01
Family and social quality of life	19.23	2.159	27.4711	3.72173	0.02
Public health quality	17.74	3.063	23.9587	4.27082	0.012

### Predictors of quality of life for parents with autism

Multiple regressions were used to predict the association of the sociodemographic characteristics of the parents with the five domains of QoL and the finding was significant (F = 3.7; p = 0.001). Table 3 summarizes the outcomes of the multiple regression tests. There is a significant negative relationship between level of education and employment status and the public health quality domain of the parents' QoL (B = -0.285, p = 00.003; B = -0.239; p = 0.026, respectively). In addition, there is a negative relationship between the parents' living condition and the quality of mental health domain (B = -0.276; p = 00.001). On the other hand, there is a positive relationship between the gender and the educational level of the parents and their quality of time management of work (B = 0.251; p = 0.013, B = 0.363; p = 0.000). As regards the quality of emotions and the family and social quality domains of the parents' QoL, no significant relationships with sociodemographic characteristics were found.

**Table 3.** Predictors for the five domains of quality of life of parents of children diagnosed with autism spectrum disorder.

Quality domain	ASD			Non-ASD		
	(B)	(Beta)	Significance	(B)	(Beta)	Significance
**Public health quality (n = 241)**						
Age (years)	0.055	0.099	0.305	0.009	0.015	0.875
Gender	-0.797	-0.124	0.228	0.210	0.030	0.747
Education level	-1.473	-0.285	0.003	2.667	0.286	0.004
Marital status	-0.587	-0.039	0.663	-0.178	-0.007	0.945
Living condition	0.965	0.049	0.586	1.222	0.230	0.022
Employment status	-10.069	0.239	0.026	1.34	0.23	0.34
**Family and social quality of life (n = 241)**						
Age (years)	0.003	0.007	0.949	0.066	0.124	0.189
Gender	0.319	0.070	0.527	2.848	0.329	0.001
Educational level	-0.164	-0.045	0.659	0.598	0.099	0.296
Marital status	0.017	0.002	0.987	0.907	0.112	0.251
Living condition	-0.614	-0.044	0.649	5.739	0.242	0.012
Employment status	-0.501	-0.159	0.169	-0.015	-0.003	0.974
**Quality of emotions (n = 241)**						
Age (years)	-0.009	-0.021	842	0.057	0.101	0.281
Gender	0.033	0.007	0.952	2.808	0.305	0.001
Educational level	-0.729	-0.183	0.073	1.487	0.232	0.015
Marital status	-0.485	-0.042	0.664	1.298	0.150	0.02
Living condition	-1.398	-0.092	0.342	3.385	0.134	0.157
Employment status	-0.463	-0.134	0.345	0.298	0.061	0.543
**Quality of mental health (n = 241)**						
Age (years)	-0.019	-0.046	0.637	0.033	0.055	0.550
Gender	0.191	0.041	0.695	1.329	0.133	0.141
Educational level	-0.423	-0.113	0.242	0.323	0.046	0.614
Marital status	-0.650	-0.059	0.514	2.565	0.093	0.310
Living condition	-3.920	-0.276	0.003	2.565	0.093	0.310
Employment status	0.518	0.160	0.143	1.394	0.261	0.008
**Quality of time management at work (n = 241)**						
Age (years)	0.018	0.041	0.659	-0.061	-0.085	0.369
Gender	1.293	0.251	0.013	1.478	0.125	0.177
Educational level	1.500	0.363	0.000	0.767	0.093	0.325
Marital status	0.033	0.003	0.975	4.075	0.367	00.000
Living condition	-0.306	-0.020	0.825	5.003	0.154	0.104
Employment status	-0.234	-0.065	0.529	1.200	0.190	0.059

ASD: Autism spectrum disorder.

## Discussion

This is one of the first studies that compared the differences in the quality of life between parents who have ASD child in comparison to parents who have not ASD child. The present study investigated the QoL of Jordanian parents who are caring for their autistic children as well as the potential associated factors of age, gender, educational level, marital status, living condition and employment status. The results showed that 66.1% (n = 80) of the mothers were involved in caregiving, which could be since these mothers were unemployed or informally employed and therefore had more time to spend with their children at home. Existing evidence suggests that maternal QoL is more affected than paternal QoL due to differences in gender coping and the nature of the child caring role that is assumed by the mothers of children with ASD [33]. The results also revealed that QoL was low among parents aged between 40 and 49 years.

The present study confirms the results in Perumal *et al.*, which reported that parents of ASD children perceived significantly lower QoL in comparison to parents of healthy children and parents of children with physical disabilities. Similarly, Taylor found that parents of children with autism and disabilities have poorer QoL due to the emotional, physical, social and financial stresses that parents experience while caring for their children. Likewise, Hoefman *et al.* [34] reported similar findings and stated that many parents experience considerable problems in combining daily activities with care, have financial problems or suffer from depressive mood and restriction on their ability to work outside the home.

In addition, when the total mean scores of QoL were analyzed across the five domains, the results suggested that there was higher impairment in the quality of mental health (mean = 21.71), quality of emotions (mean = 21.06) and the quality of time management of work (mean = 20.35) as compared with the other two domains of quality of family and social life, and public health quality. There are several explanations for poorer parental QoL in these domains. For example, the QoL impairment could be because parents of children with autism experience greater anxiety and tension as they spend longer periods of time with their children and may lack access to the appropriate services that they need to help them to cope with the difficulties they face. The impairment score for the quality of mental health domain was high because the parents of children with autism felt anxiety, were emotionally unbalanced and felt very nervous. The score for impairment in the quality of emotions domain was also high because parents of children felt nervous, were sad without any apparent reason and were psychologically lonely. In addition, one of the reasons for the perceived impairment of the quality of time management of work was that the parents were not interested in doing any activities in their free time.

In the present study, multiple regression analysis was also performed to measure whether sociodemographic factors had any significant association with the QoL domains. However, it was found that level of education and employment status contributed significantly to parental public health quality. It was also found that gender and level of education significantly contributed to poorer quality of time management of work, and that living condition significantly contributed to poorer parental mental health quality. It was therefore concluded that the sociodemographic factors of gender, living condition, level of education and employment status were the factors that were more likely to be associated with poorer parental QoL while caring for a child with ASD and that such parents were more likely to experience a negative impact on their public health quality, their mental health quality and their time management of work. These findings are consistent with Khanna *et al.* [35] which found that the mental status of parents is negatively associated with the family functioning role, and therefore lower QoL as compared with the general population.

When considering the results of the present study, the following limitations should be borne in mind. First, this study was limited to a small sample size. This is due to the recruitment of the sample from three centers and clinics that provide services to children with autism. Second, the instrument that was used was not specific to ASD. Third and finally, a comparative design was used, which is valuable for demonstrating associations, but not for drawing conclusions regarding unidirectional causative relationships. Moreover, this study did not include contributing factors that found in the literature such as poverty and lifestyle.

### Implication for the practice

This study provides evidence for the impact of having on the quality of the life of the parents which may enhance the decision make to enhance their quality of life by providing counseling services to parents and parents of children with ASD. One of the best-known social remedies to depression and the other related depressive disorders is counseling. Counseling would assist the victims to overcome the negative thoughts associated with the challenges they face in caring for children with ASD. Advocating for the public to embrace education. This concept will help to raise citizens with better skills on how to handle such challenges as caring for children with autism. Moreover, providing essential support services to parents and parents who have children with autism. One of the causes of depression and poor quality of life among parents and parent of children with autism was identified to be informal employment, which is related to financial stability, job security and satisfaction. Therefore, the responsible government bodies or other non-government social support groups need to provide social support services to help parents and parents of children with ASD.

## Conclusion

This study showed that the QoL of parents who are caring for their children with ASD was affected considerably and in a variety of ways. Jordanian parents often experienced problems with combining care with other daily living activities, had financial difficulties or suffered from depressive symptoms such as anxiety. Despite the use of a comparative design to explore parental QoL and its associated factors, the results were somewhat surprising. Hence, there is a need for further research on larger, representative samples of parents who provide direct and indirect care to children with ASDs. We also recommend conducting a longitudinal study to better understand the relationship between the characteristics of children with ASD and parental QoL.
